# Unraveling Cefiderocol Resistance in NDM- and OXA-48-like Co-Producing *Klebsiella pneumoniae* Isolates Through Integrated Genomic and Phenotypic Analysis

**DOI:** 10.3390/antibiotics15050513

**Published:** 2026-05-19

**Authors:** Simone Ambretti, Raul Cetatean, Benedetta Secci, Jessica Landi, Alessia Cantiani, Claudio Foschi

**Affiliations:** 1 Microbiology Unit, IRCCS Azienda Ospedaliero-Universitaria di Bologna, Via Massarenti 9, 40138 Bologna, Italy; simone.ambretti@aosp.bo.it (S.A.); benedetta.secci@aosp.bo.it (B.S.); 2 Department of Medical and Surgical Sciences, University of Bologna, Via Massarenti 9, 40138 Bologna, Italy; jessica.landi2@unibo.it (J.L.); alessia.cantiani@studio.unibo.it (A.C.); 3 Department of Public Health, Experimental and Forensic Medicine, University of Pavia, 27100 Pavia, Italy; raul.cetatean2@unibo.it

**Keywords:** *Klebsiella pneumoniae*, carbapenemase, NDM, OXA-48, WGS, cefiderocol, aztreonam/avibactam

## Abstract

Background/Objectives: The co-production of New Delhi metallo-β-lactamases (NDM) and OXA-48-like carbapenemases in *Klebsiella pneumoniae* represents a major therapeutic challenge due to extensive drug resistance and limited treatment options. This study aimed to investigate the molecular epidemiology, resistance profiles, and mechanisms associated with reduced susceptibility to cefiderocol in clinical isolates co-producing NDM and OXA-48-like carbapenemases. Methods: A total of 45 clinical *K. pneumoniae* isolates collected in healthcare settings in Northern Italy were analyzed. Antimicrobial susceptibility testing, including cefiderocol and aztreonam/avibactam, was performed according to EUCAST guidelines. Whole-genome sequencing was used to characterize sequence types, resistance determinants, virulence factors, plasmid replicons, and phylogenetic relationships. Mutations in iron uptake and transport genes were investigated in cefiderocol-resistant isolates. Results: Most isolates belonged to the high-risk clone ST147 (44/45) and were grouped into three main phylogenetic clusters. The isolates exhibited extensive multidrug resistance, with universal susceptibility only for aztreonam/avibactam. Cefiderocol resistance was observed in 42.2% of isolates and was unevenly distributed across the phylogeny. Mutations in iron uptake genes, particularly *cirA* and *chrA*, were identified in the majority of resistant isolates, although several strains retained wild-type sequences, indicating heterogeneous resistance mechanisms. Comparative phylogenetic analysis demonstrated close relatedness to international isolates, suggesting the global dissemination of related lineages. Conclusions: NDM- and OXA-48-like carbapenemase co-producing *K. pneumoniae* isolates are characterized by clonal dissemination, complex resistance profiles, and emerging cefiderocol resistance driven by multifactorial mechanisms. The preserved activity of aztreonam/avibactam highlights its potential as a key therapeutic option against these high-risk pathogens.

## 1. Introduction

Carbapenem-resistant *Klebsiella pneumoniae* (CRKP) represents a major global public health concern, being associated with high morbidity and mortality rates and limited therapeutic options [1]. Among resistance mechanisms, the production of carbapenemases is the most clinically relevant, with New Delhi metallo-β-lactamases (NDM) and OXA-48-like enzymes increasingly reported worldwide [2]. The co-production of NDM and OXA-48-like in *K. pneumoniae* is particularly concerning, as it confers resistance to a broad spectrum of β-lactams, including carbapenems, and severely limits the effectiveness of currently available antimicrobial agents [3,4].

In recent years, the epidemiology of carbapenemase-producing Enterobacterales has evolved toward the emergence of strains harboring multiple carbapenemase genes. These multidrug-resistant organisms are frequently associated with healthcare settings, where they can spread rapidly through clonal expansion and horizontal gene transfer mediated by mobile genetic elements such as plasmids. The co-existence of different carbapenemase genes within the same isolate may enhance bacterial adaptability and persistence, further complicating infection control and antimicrobial stewardship strategies [2,5].

The introduction of novel antimicrobial agents, such as cefiderocol (CFD) and aztreonam/avibactam, has expanded the therapeutic options against infections caused by multidrug-resistant Gram-negative bacteria, including NDM-producing strains. However, the activity of these agents against isolates co-producing NDM and OXA-48-like carbapenemases remains incompletely understood, and resistance to cefiderocol has already been reported, often involving complex and not fully elucidated mechanisms [6,7]. In particular, alterations in iron uptake systems have been proposed as contributors to cefiderocol resistance, although their role has not been consistently observed across different studies. In addition, recent studies have demonstrated that NDM-1 can hydrolyze cefiderocol, thereby contributing to reduced susceptibility in NDM-producing isolates [8].

Whole-genome sequencing (WGS) has become an essential tool for the molecular characterization of resistant pathogens, enabling high-resolution analysis of clonal relationships, resistance determinants, virulence factors, and plasmid content. This approach allows for the identification of transmission clusters and provides insights into the genetic mechanisms underlying the dissemination of high-risk clones in both clinical and colonization settings [9].

In this study, we performed a comprehensive genomic and phenotypic characterization of 45 *K. pneumoniae* isolates co-producing NDM and OXA-48-like carbapenemases collected in healthcare settings in the metropolitan area of Bologna, Italy. By integrating antimicrobial susceptibility testing, including cefiderocol and aztreonam/avibactam, with WGS-based analyses, we aimed to (i) investigate the molecular epidemiology of these isolates, (ii) characterize their resistance and virulence profiles, and (iii) explore potential mechanisms associated with reduced susceptibility to cefiderocol.

## 2. Results

### 2.1. Phenotypic Analysis

The isolates exhibited a multidrug-resistant phenotype, with high resistance rates observed across most of the tested antibiotics.

Resistance to ampicillin, amoxicillin/clavulanate, ciprofloxacin, piperacillin/tazobactam, and ertapenem was detected in all isolates (45/45, 100%). Similarly, very high resistance rates were observed for cefepime, ceftazidime, ceftazidime/avibactam, ceftolozane/tazobactam, cefotaxime, and meropenem (44/45, 97.8% for each antibiotic). Resistance to gentamicin was also frequent (41/45, 91.1%), while susceptibility to amikacin was retained in a minority of isolates (10/45, 22.2%).

Resistance to colistin was observed in 9/45 isolates (20.0%), while resistance to tigecycline was detected in 16/45 isolates (35.6%), further highlighting the limited therapeutic options against these multidrug-resistant strains.

Regarding novel antimicrobial agents, 19/45 isolates (42.2%) were resistant to cefiderocol, with MIC values of 4 mg/L in 17/19 (89.5%) isolates and 8 mg/L in 2/19 (10.5%). The MIC_50_ and MIC_90_ were 2 and 4 mg/L, respectively, with a median MIC (IQR) of 2 (2–4) mg/L. In contrast, all isolates (45/45, 100%) were susceptible to aztreonam/avibactam, with MIC_50_ and MIC_90_ values of 0.25/4 mg/L and a median MIC (IQR) of 0.25 (0.25–0.25) mg/L.

A detailed summary of antimicrobial susceptibility profiles is provided in Supplementary Table S1.

### 2.2. Genomic Characterization of the Isolates

In silico MLST analysis assigned 44/45 isolates to sequence type ST147, including two single-locus variants (ST147-1LV), while one isolate (MBL46) belonged to ST11. Capsule (K) and O-antigen typing revealed that the majority of isolates carried the KL64 capsule locus and O2a O-antigen (36/45), followed by KL10/O3–O3a (7/45). The remaining isolates belonged to KL51/O3–O3a (MBL15) and KL24/O2a (MBL46) (Supplementary File S1).

**Figure 1:**
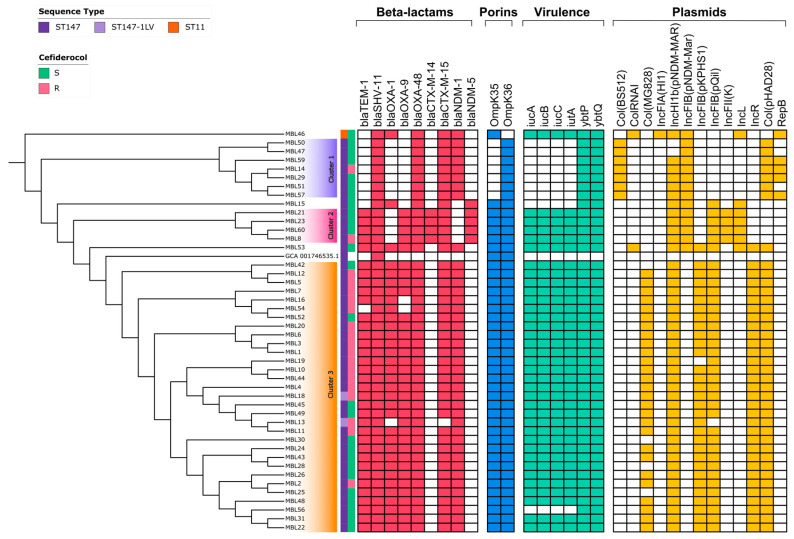
**Core genome phylogenetic tree of the study isolates.** Colored blocks indicate the three major phylogenetic clusters identified in the dataset. The accompanying heatmap reports, from left to right, sequence type (ST), cefiderocol susceptibility phenotype, β-lactam resistance genes, porin alterations, virulence determinants, and plasmid replicons. Colored cells indicate the presence of the corresponding feature, whereas empty cells indicate absence. Cefiderocol-resistant isolates are highlighted according to EUCAST breakpoints. The distribution of resistance and virulence determinants across the phylogeny illustrates the cluster-specific genomic profiles of the isolates.

Core genome phylogenetic analysis identified three major clusters among the isolates (Figure 1). The distribution of resistance determinants, virulence factors, and plasmid replicons across the phylogenetic tree revealed cluster-specific genomic signatures, highlighting the strong association between clonal lineage and accessory genome composition (Figure 1).

Cluster 1 comprised seven isolates, all belonging to KL10 and O3/O3a loci, and displaying highly conserved antimicrobial resistance and virulence gene profiles. All isolates harbored the same β-lactam resistance genes (*blaNDM-1*, *blaSHV-11*, *blaOXA-48*, *blaCTX-M-15*).

Regarding outer membrane porins, all strains carried an in-frame insertion in OmpK36 (D135DGD), while no mutations were detected in OmpK35. Several macrolide resistance genes were identified, including *msr(E)*, *mph(A)*, *mph(E)*, *mrx(A)*, and *arm(A)*. Point mutations in quinolone resistance-determining regions were observed in *gyrA* (S83Y, D87A/N) and *parC* (S80I), while no *qnr* genes were detected. No genes conferring phenicol or rifamycin resistance were identified; however, *tet(A)*, conferring tetracycline resistance, was present exclusively in this cluster.

Virulence analysis identified only the yersiniabactin locus, with Kleborate assigning a virulence score of 1 to all isolates. The complete yersiniabactin locus (YbST 78, lineage 10) was detected and associated with ICEKp4. No aerobactin, salmochelin, colibactin, or hypermucoviscosity-associated genes (*rmpA*, *rmpA2*) were identified. Notably, cluster 1 isolates uniquely carried mercury resistance genes (*merACDEPRT*).

Plasmid analysis revealed a conserved profile, including Col(BS512), IncFIB(pNDM-Mar), Col(pHAD28), and repB(R1701).

Pairwise SNP distance analysis showed a range of 3–57 SNPs, indicating moderate genomic diversity. Two isolate pairs (MBL47–MBL50 and MBL51–MBL57) exhibited particularly low SNP distances (3 and 14 SNPs, respectively), consistent with recent transmission events, while the remaining isolates showed greater divergence.

Cluster 2 included four isolates with highly conserved genomic features, consistent with clonal relatedness (0–11 SNPs). All isolates carried *blaTEM-1*, *blaSHV-11*, *blaOXA-9*, *blaOXA-48*, *blaCTX-M-14*, *blaCTX-M-15*, and *blaNDM-5* (Supplementary File S1). Additional resistance determinants included the 16S rRNA methyltransferase gene *rmtF1*, quinolone resistance genes *qnrB1* and *qnrS1*, and the rifamycin resistance gene *arr-2.* A frameshift mutation in OmpK35 (F300Lfs*25), resulting in a premature stop codon, was identified in all isolates. The in-frame insertion in OmpK36 observed in cluster 1 was also present in this cluster. Additionally, the acid resistance protein Asr was detected.

Virulence profiling revealed the presence of both aerobactin and yersiniabactin loci, with a Kleborate virulence score of 4. The complete aerobactin locus (AbST 63, lineage 1) and yersiniabactin locus (YbST 584, lineage 9) were identified. The hypermucoviscosity determinant *rmpA2* was truncated in all isolates. No salmochelin or colibactin loci were detected.

Plasmid replicons were conserved and included IncHI1b(pNDM-MAR), IncFIB(pNDM-Mar), IncFIB(pQil), IncFII(K), and IncL.

Cluster 3 was the largest group, comprising 31 isolates with highly similar resistance profiles. All isolates carried *blaNDM-1*, *blaOXA-48*, and *blaSHV-11*, while *blaTEM-1*, *blaOXA-1*, and *blaCTX-M-15* were detected in 30/31 isolates and *blaOXA-9* in 29/31 isolates.

No macrolide resistance genes were identified in this cluster. All isolates harbored the yersiniabactin locus, while the aerobactin locus was present in 30/31 isolates, with one exception (MBL56). The complete yersiniabactin locus (lineage 9, ICEKp3) was identified in all isolates. Hypermucoviscosity-associated genes (*rmpA* and *rmpA2*) were present in 23/31 and 18/31 isolates, respectively, and were truncated in all cases.

Plasmid analysis revealed a conserved profile including Col(pHAD28), IncR, IncHI1b(pNDM-Mar), IncFIB(pQil), IncFIB(pKPHS1), and Col(MG828).

Pairwise SNP distances ranged from 0 to 28 SNPs, indicating a relatively tight genomic cluster. Most isolates differed by 0–20 SNPs, while MBL42 showed slightly higher divergence (15–28 SNPs).

Three isolates (MBL15, MBL46, and MBL53) did not cluster within the main groups and displayed distinct genomic features (Figure 1).

MBL46 belonged to ST11 and carried *blaSHV-11*, *blaOXA-1*, *blaOXA-48*, *blaCTX-M-15*, and *blaNDM-1.* It harbored a frameshift mutation in *mgrB* (N25Kfs*9), a well-known mechanism associated with colistin resistance, and a point mutation in *pmrB* (T140P), whose role in resistance has not been previously described. However, despite these genetic alterations, the isolate was phenotypically susceptible to colistin. This finding is consistent with previous reports showing that *mgrB* alterations may also occur in colistin-susceptible isolates, highlighting the complexity of resistance mechanisms and genotype–phenotype relationships [10]. A frameshift mutation in OmpK35 was also present, while OmpK36 remained wild-type, making this isolate unique within the dataset. Both aerobactin (AbST 95) and yersiniabactin (YbST 230, ICEKp11) loci were detected. The identified plasmids were ColRNAI, IncFIA(HI1), lncHI1b(pNDM-MAR), IncFIB(pNDM-Mar), IncL and repB(R1701).

MBL15 (ST147, KL51) carried multiple β-lactamase genes, including *blaSHV-11*, *blaOXA-1*, *blaOXA-48*, *blaCTX-M-15*, and *blaNDM-5*. Mutations were present in both OmpK35 and OmpK36. Only the yersiniabactin locus was detected. The plasmids found were: Col(BS512), lncHI1b(pNDM-MAR), IncFIB(pNDM-Mar), IncFIB(pQil) and IncL.

MBL53 (ST147, KL64) showed a resistance profile similar to cluster 3 but additionally carried macrolide resistance genes (*msr(E)*, *mph(A)*, *mph(E)*, *mrx(A)*, *arm(A)*, similar to cluster 1). A truncating mutation in *mgrB* (K34*) was also identified. Plasmid replicons identified were CorRNAI, lncHI1b(pNDM-MAR), IncFIB(pNDM-Mar), IncFIB(pKPHS1), IncFIB(pQil), IncL, IncR and Col(pHAD28).

### 2.3. Global Phylogenetic Context

**Figure 2:**
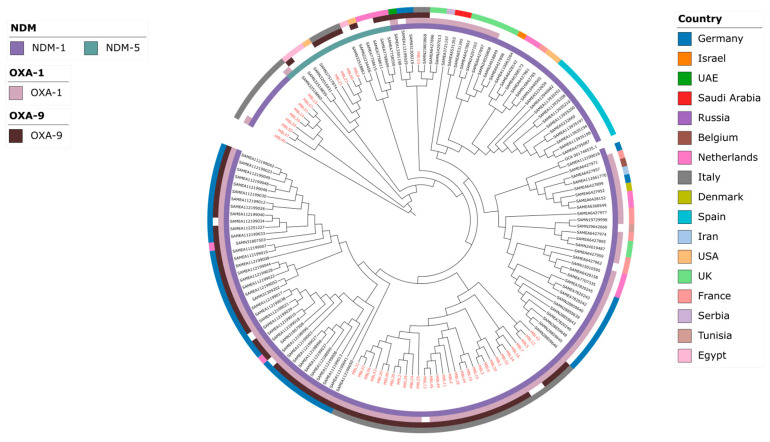
**Core genome phylogenetic analysis of *K. pneumoniae* isolates co-producing NDM-1 or NDM-5 and OXA-48.** Study isolates are highlighted in red. The concentric rings represent, from the innermost to the outermost layer, the NDM variant (NDM-1 or NDM-5), the presence of *blaOXA-1*, the presence of *blaOXA-9*, and the country of isolation. Colored sectors indicate the corresponding feature or geographic origin, whereas empty sectors indicate absence of the corresponding determinant. Cefiderocol susceptibility data were not available for publicly retrieved genomes and were therefore not included in the analysis. The phylogeny highlights the international distribution and genetic relatedness of the analyzed isolates.

Comparative phylogenetic analysis including 109 publicly available genomes co-producing NDM and OXA-48 revealed that isolates included in this study were interspersed among publicly available genomes from multiple geographic regions, including Europe, the United States, and the Middle East/North Africa (Figure 2). This pattern indicates close genetic relatedness among geographically diverse isolates and supports the hypothesis of international dissemination of related lineages rather than local, geographically restricted evolution.

In addition, the presence of multiple sublineages within ST147 suggests the coexistence of distinct evolutionary branches, potentially reflecting multiple introduction events or ongoing diversification within this high-risk clone.

The distribution of additional β-lactamase genes, such as OXA-1 and OXA-9, varied across the phylogeny, further highlighting the heterogeneity of the accessory resistome among closely related isolates.

### 2.4. Analysis of Iron Uptake and Transport Genes

To investigate potential mechanisms underlying cefiderocol resistance, amino acid sequences of genes involved in iron uptake and transport were extracted and aligned against those of *K. pneumoniae* KP-1PI (accession number CP071027), a cefiderocol-susceptible reference strain [11,12].

Mutations in iron uptake and transport genes were identified in 16/19 isolates exhibiting cefiderocol resistance (MIC 4–8 mg/L). None of the mutations identified in iron uptake and transport genes were detected among cefiderocol-susceptible isolates.

The most frequently observed alterations were in *cirA* (D654G in 5/19 isolates) and *chrA* (I128V in 4/19 isolates and K133N in 3/19 isolates), among others (Table 1). The D654G substitution in *cirA* was significantly associated with increased cefiderocol MICs (Fisher’s exact test, *p* = 0.0095; odds ratio [OR] = ∞, 95% CI: 1.43–∞). The I128V substitution in *chrA* showed a weaker association (*p* = 0.026; OR = ∞, 95% CI: 0.98–∞), while the K133N substitution did not reach statistical significance (*p* = 0.068; OR = ∞, 95% CI: 0.59–∞). Odds ratios of ∞ reflect complete separation in the dataset due to the absence of these mutations among isolates with lower MIC values.

None of the missense mutations detected have been previously reported in the literature. In addition to genes involved in iron uptake and transport, the gene encoding penicillin-binding protein 3 (PBP3; *ftsI*) was also systematically analyzed, given its reported association with reduced susceptibility to cefiderocol [12]. No mutations or amino acid substitutions were identified in *ftsI* in any of the isolates.

Notably, mutations were not detected in all cefiderocol-resistant isolates, as several strains (e.g., MBL6, MBL11, MBL14, MBL16, and MBL19) retained wild-type sequences in all analyzed genes. In some isolates, multiple alterations affecting different iron transport genes were observed, particularly in strains with higher MIC values.

Consistently, cefiderocol resistance was not evenly distributed across the phylogeny (Figure 1), suggesting that reduced susceptibility is linked to specific genetic backgrounds rather than solely to carbapenemase production. Accordingly, the co-occurrence of NDM-1 or NDM-5 with OXA-48 did not fully account for the observed resistance phenotype, supporting the involvement of additional or alternative mechanisms.

To further investigate the mechanisms underlying cefiderocol resistance, copy number analysis of *blaNDM-1* and *blaNDM-5* genes was performed, given previous reports linking increased NDM abundance to reduced susceptibility [13].

The analysis showed that all isolates carried a single copy of the *blaNDM* gene, based on sequencing read depth normalized to chromosomal coverage. Overall, no consistent increase in *blaNDM* copy number was observed among cefiderocol-resistant isolates compared to susceptible ones, indicating that gene dosage alone is unlikely to account for the observed variability in CFD susceptibility.

## 3. Discussion

Carbapenem-resistant *Klebsiella pneumoniae* represents a major clinical challenge, particularly when multiple carbapenemase genes co-occur within the same isolate. In this study, we provide a comprehensive genomic and phenotypic characterization of NDM- and OXA-48-like co-producing *K. pneumoniae* isolates collected in a healthcare setting, highlighting both the clonal structure of circulating strains and the complexity of resistance mechanisms to novel antimicrobial agents.

A key finding of our study is the predominance of the high-risk clone ST147, which accounted for the vast majority of isolates. This observation is consistent with previous reports identifying ST147 as a globally disseminated lineage associated with multidrug resistance and epidemic spread [1,5]. Core genome phylogenetic analysis further revealed the presence of multiple closely related clusters, suggesting ongoing transmission within the healthcare setting. At the same time, comparative analysis with publicly available genomes demonstrated that our isolates were interspersed among strains from different geographic regions, supporting the hypothesis of international dissemination rather than geographically confined evolution [1,5]. The presence of multiple sublineages within ST147 further suggests either repeated introduction events or local diversification of this high-risk clone.

However, the lack of phenotypic data (e.g., cefiderocol susceptibility) for publicly available genomes limited the ability to directly correlate phylogenetic clustering with resistance profiles.

From a phenotypic perspective, the isolates displayed an extensively drug-resistant profile, with resistance to most β-lactams and other commonly used antimicrobial agents. Notably, ceftazidime/avibactam was largely ineffective, consistent with the presence of metallo-β-lactamases, while aztreonam/avibactam retained full in vitro activity against all isolates. This finding reinforces the potential role of aztreonam/avibactam as a key therapeutic option for infections caused by MBL-producing Enterobacterales, including those co-producing OXA-48-like carbapenemases [6].

In contrast, reduced susceptibility to cefiderocol was observed in a substantial proportion of isolates (42.2%), highlighting the emergence of resistance to this last-resort agent. Importantly, cefiderocol resistance was not uniformly distributed across the phylogeny, suggesting that specific genetic backgrounds may influence susceptibility.

This observation is in line with the increasing recognition that cefiderocol resistance is multifactorial and cannot be solely attributed to the presence of carbapenemase genes [12]. In addition, recent evidence suggests that NDM enzymes may directly hydrolyze cefiderocol, thereby contributing to reduced susceptibility in NDM-producing isolates [8]. Although we did not observe an association with *blaNDM* copy number in our dataset, this mechanism may still contribute to resistance in combination with other factors.

Our analysis of iron uptake and transport systems identified mutations in the majority of cefiderocol-resistant isolates, particularly in *cirA* and *chrA*, supporting a potential role of these pathways in reduced susceptibility. However, these alterations were not consistently observed across all resistant isolates, as several strains retained wild-type sequences in all analyzed genes. Furthermore, the presence of multiple mutations in some isolates, contrasted with their absence in others with similar MIC values, suggests that no single mutational pattern fully explains the resistance phenotype. These findings are consistent with previous reports, including the study by Coppi et al. [14], which demonstrated that cefiderocol resistance can occur in the absence of detectable alterations in iron uptake genes, further supporting the hypothesis of heterogeneous and multifactorial resistance mechanisms.

Given the siderophore-mediated mechanism of cefiderocol uptake, alterations in iron acquisition systems may affect intracellular antibiotic accumulation. Variability in the expression or functionality of siderophore receptors could therefore contribute to the heterogeneous susceptibility patterns observed, even in the absence of consistent mutational profiles [12].

Notably, no mutations were identified in the *ftsI* gene encoding PBP3, despite previous reports linking PBP3 alterations to reduced cefiderocol susceptibility. This finding suggests that, in our dataset, PBP3 does not play a major role in the observed resistance phenotype, further supporting the heterogeneity of the underlying mechanisms.

In addition, the possibility of heteroresistance cannot be excluded, as previously described for cefiderocol. Subpopulations with reduced susceptibility may not be fully captured by standard MIC testing, potentially contributing to the observed variability in phenotypic resistance [12].

Beyond iron transport systems, additional factors may contribute to cefiderocol resistance, including alterations in outer membrane permeability, efflux pump activity, and changes affecting antibiotic uptake, as previously suggested [12]. In our dataset, porin alterations, such as the insertion in OmpK36 and mutations in OmpK35, were frequently observed and may play a contributory role, although their direct impact on cefiderocol susceptibility remains to be fully elucidated [15].

The analysis of virulence determinants and plasmid content further highlighted the heterogeneity of accessory genomes across clusters. While cluster 1 isolates were characterized by a limited virulence repertoire, clusters 2 and 3 frequently harbored both aerobactin and yersiniabactin loci, suggesting differences in virulence potential among closely related strains. Similarly, cluster-specific plasmid profiles indicate that horizontal gene transfer plays a major role in shaping the resistome and mobilome of these pathogens [5].

This study has several limitations that should be acknowledged. First, the use of short-read sequencing precluded the generation of complete chromosome-level assemblies, limiting the precise reconstruction of plasmid structures and the genomic contexts of resistance genes. Second, the functional impact of the identified missense mutations on cefiderocol susceptibility was not experimentally validated; therefore, their direct contribution to the observed resistance phenotype could not be definitively established. Further studies, including functional assays and transcriptomic analyses, are needed to clarify the role of these mutations. Additionally, the lack of confirmatory analyses (e.g., qPCR or Western blotting) precludes direct assessment of *blaNDM* gene dosage and expression. Therefore, the potential contribution of increased NDM production to cefiderocol resistance should be interpreted with caution.

In conclusion, our findings highlight the complex interplay between clonal dissemination, accessory genome composition, and multifactorial resistance mechanisms in NDM- and OXA-48-like co-producing *K. pneumoniae*. The emergence of cefiderocol resistance, even in the absence of consistent genetic determinants, underscores the need for continued surveillance and a deeper understanding of resistance pathways. At the same time, the preserved activity of aztreonam/avibactam supports its potential role as a valuable therapeutic option against these highly resistant pathogens.

## 4. Materials and Methods

### 4.1. Bacterial Strains and Antimicrobial Susceptibility Testing

In this study, 45 clinical isolates of *Klebsiella pneumoniae* co-producing NDM and OXA-48-like carbapenemases were included. Isolates were collected between February 2024 and December 2025 in the metropolitan area of Bologna (Italy), including the Microbiology Unit of IRCCS Azienda Ospedaliero-Universitaria di Bologna (S. Orsola Hospital), Maggiore Hospital, and Imola Hospital.

Each isolate was assigned a unique identifier (e.g., MBL1) for study purposes, where the prefix “MBL” denotes metallo-β-lactamase-producing strains.

The isolates were recovered from different clinical specimens, including urines (21/45), blood cultures (11/45), respiratory samples (9/45), rectal swabs (2/45), biopsy (1/45), and abdominal drainage (1/45).

Species identification was performed using MALDI-TOF mass spectrometry (Bruker Daltonik GmbH, Bremen, Germany). Antimicrobial susceptibility testing was carried out using the MicroScan WalkAway-96 system (Beckman Coulter, Brea, CA, USA). The following antibiotics were tested: ceftazidime (CAZ), meropenem (MEM), amoxicillin/clavulanate (AMC), ceftazidime/avibactam (CZA), cefepime (FEP), piperacillin/tazobactam (P/T), ciprofloxacin (CIP), amikacin (AMI), gentamicin (GEN), ampicillin (AMP), ceftolozane/tazobactam (C/T), cefotaxime (CTX), ertapenem (ERT), colistin (COL), tigecycline (TGC) and trimethoprim/sulfamethoxazole (SXT). Minimum inhibitory concentrations (MICs) were interpreted according to EUCAST clinical breakpoints (version 16.0, 2026, available at: https://www.eucast.org/bacteria/clinical-breakpoints-and-interpretation/clinical-breakpoint-tables/) (accessed on 11 February 2026).

Carbapenemase production was confirmed using a multiplex immunochromatographic assay (NG-Test CARBA 5; NG Biotech, Guipry, France).

Susceptibility to cefiderocol (CFD) was determined by broth microdilution in iron-depleted medium, following EUCAST recommendations. Susceptibility to aztreonam/avibactam (AZA) was assessed using the Sensititre EUAZAXF plate (Thermo Fisher Scientific, Waltham, MA, USA).

### 4.2. Whole-Genome Sequencing and Bioinformatic Analyses

Whole-genome sequencing (WGS) was performed for genomic characterization. Genomic DNA was extracted using the DNeasy Blood & Tissue Kit (Qiagen, Hombrechtikon, Switzerland), purified with AMPure XP magnetic beads (Beckman Coulter, Brea, CA, USA), and quantified using the Qubit dsDNA BR Assay Kit (Thermo Fisher Scientific, USA). Illumina paired-end libraries were prepared using the DNA Prep Library Preparation Kit (Illumina, San Diego, CA, USA), and sequencing was carried out on Illumina platforms.

Raw paired-end reads were first screened with FastQC v.0.12.1 (https://github.com/s-andrews/FastQC, accessed on 11 February 2026). Reads were then trimmed using trim_galore v.0.6.10 with a default quality Phred score of 20 and minimum length of 50 bp (https://github.com/FelixKrueger/TrimGalore, accessed on 11 February 2026). Quality of trimmed reads was reassessed with FastQC. Potential contamination was evaluated using Kraken2 v.2.1.3 and Bracken v.3.0.1 [16,17].

Genome assembly was performed using SPAdes v. 4.0.0 [18] with the *--isolate* flag. QUAST v. 5.3.0 was then employed to evaluate the assembly metrics [19]. Genome annotation was carried out with Bakta v.1.8.1 [20]. In silico multi-locus sequence typing (MLST) was performed using the *mlst* tool against the PubMLST database (https://github.com/tseemann/mlst, accessed on 11 February 2026) [21].

Capsule (K) and O-antigen loci, as well as acquired antimicrobial resistance genes, were identified using Kleborate v.3.1.2 with the *klebsiella_pneumo_complex__kaptive* and *klebsiella_pneumo_complex__amr* modules [22,23]. Virulence profiling was also performed using Kleborate, including the identification of virulence loci and calculation of virulence scores. In parallel, antimicrobial resistance genes, virulence determinants, and point mutations were identified using AMRFinderPlus v.4.2.5 (database version 21 January 2026) [24]. Plasmid replicons were identified using PlasmidFinder v.2.1 [25]. All bioinformatic analyses were automated using a custom Snakemake workflow [26].

Core genome phylogenetic analysis was performed using ParSNP v.2.1.1 [27], with *K. pneumoniae* strain TGH13 (accession number GCA_001746535.1) as the reference genome. This reference was selected because it belonged to the same sequence type and was available as a complete genome. To provide broader genomic context, 2539 *K. pneumoniae* ST147 genomes carrying KL64, KL51, or KL10 loci were retrieved from Pathogenwatch platform (https://pathogen.watch/, accessed on 11 February 2026). Genomes co-harboring NDM and OXA-48 were selected, resulting in a dataset of 109 publicly available genomes included in the comparative phylogenetic analysis. The sequence type distribution of the publicly available genomes was also assessed, confirming that all isolates belonged to ST147.

A second phylogenetic tree, including only study isolates and the reference genome, was also generated. Metadata for the publicly available genomes are reported in Supplementary File S2.

Phylogenetic trees were visualized using iTOL v.7.

Variant calling was performed using Snippy v.4.6.0 with the *snippy-multi* pipeline, using the same reference genome. Recombinant regions were filtered using Gubbins v.3.4 [28], and pairwise SNP distances were calculated using snp-dists v.0.8.2.

To investigate potential molecular mechanisms behind Cefiderocol resistance, iron uptake/transport genes were manually investigated by extracting the amino acid sequences from the study isolates and aligning them against sequences extracted from the complete genome of *K. pneumoniae* KP-1PI (accession number: CP071027), a CFD-susceptible strain isolated from an outbreak in Tuscany, Italy, in 2018. The alignment was performed with MAFFT version 7.526 using the auto flag [29].

Copy number analysis of *blaNDM* genes was performed using the carbapenemase-encoding gene copy number estimator (CCNE) tool [30], which estimates gene copy number based on sequencing read depth normalized to average chromosomal coverage.

## Figures and Tables

**Table 1:** **Missense mutations identified in iron uptake and transport genes in *Klebsiella pneumoniae* cefiderocol-resistant isolates.** Cefiderocol-susceptible isolates did not harbor any of the mutations listed. WT = wild type.

		Mutations in Iron Uptake/Transport Genes				
Isolate	MIC (mg/L)	*chrA*	*cirA*	*efeO*	*fepA*	*fhuA*
MBL1	4	WT	D654G	WT	WT	WT
MBL2	4	K133N	Ins658LLPALKVRL	WT	WT	WT
MBL3	4	I128V, K133N	WT	WT	WT	T42N, A48P, S54N
MBL4	4	I128V, Y130F	WT	WT	WT	WT
MBL5	4	WT	D654G	WT	WT	WT
MBL6	4	WT	WT	WT	WT	WT
MBL7	4	I128V, Y130F	WT	WT	WT	WT
MBL8	4	K133N	D654G	WT	WT	WT
MBL10	4	I128V	WT	WT	WT	WT
MBL11	4	WT	WT	WT	WT	WT
MBL12	4	WT	D654G	WT	WT	WT
MBL13	8	N154K	D654G, S656R	DA127EG	S456C	WT
MBL14	8	WT	WT	WT	WT	WT
MBL16	4	WT	WT	WT	WT	WT
MBL18	4	N154K	WT	WT	WT	WT
MBL19	4	WT	WT	WT	WT	WT

## Data Availability

All the relevant research data of this study can be found in the article or in the Supplementary Materials. Raw genomic data of the bacterial isolates have been deposited on on-line repositories (BioProject ID: PRJNA1448013 at NCBI, https://www.ncbi.nlm.nih.gov/bioproject/, accessed on 15 March 2026).

## Supplementary Materials

The following supporting information can be downloaded at: https://www.mdpi.com/article/10.3390/antibiotics15050513/s1, Table S1: Antimicrobial susceptibility profiles (MIC values) of *Klebsiella pneumoniae* clinical isolates included in this study. File S1: Metadata and genomic characteristics of *Klebsiella pneumoniae* isolates included in this study, including sequence type, capsule and O-antigen loci, antimicrobial resistance genes, virulence determinants, and plasmid replicons. File S2: List and metadata of publicly available *Klebsiella pneumoniae* genomes included in the comparative phylogenetic analysis, including country of origin, sequence type, and carbapenemase gene content.

## Abbreviations

AMC	Amoxicillin/Clavulanate
AMI	Amikacin
AMP	Ampicillin
AZA	Aztreonam/avibactam
CAZ	Ceftazidime
CFD	Cefiderocol
CIP	Ciprofloxacin
CRKP	Carbapenem-resistant Klebsiella pneumoniae
C/T	Ceftolozane/Tazobactam
CTX	Cefotaxime
CZA	Ceftazidime/Avibactam
ERT	Ertapenem
FEP	Cefepime
GEN	Gentamicin
MBL	metallo-β-lactamase
MEM	Meropenem
MIC	minimum inhibitory concentration
MLST	multi-locus sequence typing
P/T	Piperacillin/Tazobactam
SXT	Trimethoprim/sulfamethoxazole
WGS	Whole-genome sequencing
