# Supplementary material for: Unraveling Cefiderocol Resistance in NDM- and OXA-48-like Co-Producing Klebsiella pneumoniae Isolates Through Integrated Genomic and Phenotypic Analysis
Source: Antibiotics (Basel). 2026 May 19;15(5):513. doi: 10.3390/antibiotics15050513 (PMC13203471; doi:10.3390/antibiotics15050513)
Supplement: Supplementary file 1 [file antibiotics-15-00513-s001.zip › Supplementary Table S1.pdf]

Table S1. Antimicrobial susceptibility profiles (MIC values) of *Klebsiella pneumoniae* clinical isolates included in this study

	Antimicrobial MIC Value (mg/L)																	
Isolate	AZA	CFD	AMI	AMP	AMC	CZA	CAZ	C/T	CIP	CTX	ERT	FEP	GEN	MEM	SXT	P/T	COL	TGC
MBL1	0,5/4	4	16	>8	>32	>8	>32	>4	>1	>32	>1	>8	>4	>32	>4/76	>16	>4	>1
MBL2	0,25/4	4	16	>8	>32	>8	>32	>4	>1	>32	>1	>8	>4	>32	≤2/38	>16	≤2	≤0,5
MBL3	0,25/4	4	16	>8	>32	>8	>32	>4	>1	>32	>1	>8	>4	>32	≤2/38	>16	≤2	≤0,5
MBL4	0,25/4	4	16	>8	>32	>8	>32	>4	>1	>32	>1	>8	>4	>32	≤2/38	>16	≤2	≤0,5
MBL5	0,25/4	4	16	>8	>32	>8	>32	>4	>1	>32	>1	>8	>4	>32	≤2/38	>16	≤2	≤0,5
MBL6	0,25/4	4	16	>8	>32	>8	>32	>4	>1	>32	>1	>8	>4	>32	≤2/38	>16	>4	≤0,5
MBL7	0,25/4	4	16	>8	>32	>8	>32	>4	>1	>32	>1	>8	>4	>32	≤2/38	>16	≤2	≤0,5
MBL8	0,25/4	4	>16	>8	>32	>8	>32	>4	>1	>32	>1	>8	>4	>32	>4/76	>16	≤2	1
MBL10	0,25/4	4	16	>8	>32	>8	>32	>4	>1	>32	>1	>8	>4	>32	≤2/38	>16	≤2	≤0,5
MBL11	0,25/4	4	>16	>8	>32	>8	>32	>4	>1	>32	>1	>8	>4	>32	≤2/38	>16	≤2	≤0,5
MBL12	0,25/4	4	16	>8	>32	>8	>32	>4	>1	>32	>1	>8	>4	>32	≤2/38	>16	≤2	1
MBL13	0,25/4	8	16	>8	>32	>8	>32	>4	>1	>32	>1	>8	>4	>32	≤2/38	>16	≤2	≤0,5
MBL14	0,25/4	8	>16	>8	>32	>8	>32	>4	>1	>32	>1	>8	>4	>32	>4/76	>16	≤2	≤0,5
MBL15	0,25/4	2	>16	>8	>32	>8	>32	>4	>1	>32	>1	>8	>4	>32	>4/76	>16	≤2	>1
MBL16	0,25/4	4	16	>8	>32	>8	>32	>4	>1	>32	>1	>8	>4	>32	≤2/38	>16	≤2	1
MBL18	0,25/4	4	≤8	>8	>32	>8	>32	>4	>1	>32	>1	>8	>4	>32	≤2/38	>16	≤2	≤0,5
MBL19	0,25/4	4	16	>8	>32	≤2	≤0,5	≤0,5	>1	2	>1	1	>4	8	≤/38	>16	≤2	1
MBL20	0,25/4	4	16	>8	>32	>8	>32	>4	>1	>32	>1	>8	>4	>8	≤2/38	>16	≤2	≤0,5
MBL21	0,25/4	2	16	>8	>32	>8	>32	>4	>1	>32	>1	>8	>4	>32	≤2/38	>16	≤2	≤0,5
MBL22	0,25/4	2	16	>8	>32	>8	>32	>4	>1	>32	>1	>8	>4	>8	>4/76	>16	≤2	≤0,5
MBL23	0,25/4	2	>16	>8	>32	>8	>32	>4	>1	>32	>1	>8	>4	>8	>4/76	>16	>4	>0,5
MBL24	0,25/4	2	16	>8	>32	>8	>32	>4	>1	>32	>1	>8	>4	>8	≤2/38	>16	≤2	≤0,5
MBL25	0,24/4	2	≤8	>8	>32	>8	>32	>4	>1	>32	>1	>8	>4	>8	≤2/38	>16	≤2	>0,5
MBL26	0,25/4	2	<8	>8	>32	>8	>32	>4	>1	>32	>1	>8	>4	>32	>4/76	>16	≤2	≤0,5
MBL28	0,25/4	2	≤8	>8	>32	>8	>32	>4	>1	>32	>1	>8	>4	>8	≤2/38	>16	≤2	≤0,5

MBL29	0,25/4	1	>16	>8	>32	>8	>32	>4	>1	>32	>1	>8	>4	>32	>4/76	>16	≤2	1
MBL30	0,25/4	2	≤8	>8	>32	>8	>32	>4	>1	>32	>1	>8	>4	>8	≤2/38	>16	≤2	>0,5
MBL31	0,25/4	2	16	>8	>32	>8	>32	>4	>1	>32	>1	>8	>4	>8	>4/76	>16	≤2	≤0,5
MBL42	0,25/4	2	16	>8	>32	>8	>32	>4	>1	>32	>1	>8	>4	>8	≤2/38	>16	>4	≤0,5
MBL43	0,25/4	2	≤8	>8	>32	>8	>32	>4	>1	>32	>1	>8	>4	>32	>4/76	>16	≤2	≤0,5
MBL44	0,12/4	4	16	>8	>32	>8	>32	>4	>1	>32	>1	>8	>4	>32	<2/38	>16	≤2	≤0,5
MBL45	0,25/4	2	16	>8	>32	>8	>32	>4	>1	>32	>1	>8	>4	>8	>4/76	>16	≤2	>0,5
MBL46	0,25/4	2	16	>8	>32	>8	>32	>4	>1	>32	>1	>8	>4	32	>4/76	>16	≤2	≤0,5
MBL47	0,25/4	1	≤8	>8	>32	>8	>32	>4	>1	>32	>1	>8	<=2	>8	≤2/38	>16	>4	>0,5
MBL48	0,25/4	2	≤8	>8	>32	>8	>32	>4	>1	>32	>1	>8	>4	>8	≤2/38	>16	4	≤0,5
MBL49	0,25/4	2	≤8	>8	>32	>8	>32	>4	>1	>32	>1	>8	>4	>8	≤2/38	>16	≤2	≤0,5
MBL50	0,25/4	1	≤8	>8	>32	>8	>32	>4	>1	>32	>1	>8	<=2	>8	≤2/38	>16	≤2	>0,5
MBL51	0,25/4	1	>16	>8	>32	>8	>32	>4	>1	>32	>1	>8	>4	>8	>4/76	>16	≤2	>0,5
MBL52	0,25/4	2	16	>8	>32	>8	>32	>4	>1	>32	>1	>8	<=2	>32	≤2/38	>16	≤2	≤0,5
MBL53	0,25/4	2	>16	>8	>32	>8	>32	>4	>1	>32	>1	>8	>4	>8	>4/76	>16	>4	>0,5
MBL54	0,12/4	4	16	>8	>32	>8	>32	>4	>1	>32	>1	>8	<=2	>32	≤2/38	>16	>4	≤0,5
MBL56	0,25/4	2	>16	>8	>32	>8	>32	>4	>1	>32	>1	>8	>4	>8	≤2/38	>16	≤2	≤0,5
MBL57	0,25/4	1	>16	>8	>32	>8	>32	>4	>1	>32	>1	>8	>4	>8	>4/76	>16	≤2	>0,5
MBL59	0,25/4	1	>16	>8	>32	>8	>32	>4	>1	>32	>1	>8	>4	>8	>4/76	>16	4	>0,5
MBL60	0,25/4	1	>16	>8	>32	>8	>32	>4	>1	>32	>1	>8	>4	>32	>4/76	>16	≤2	1

AZA, Aztreonam/Avibactam; CFD, Cefiderocol; AMI, Amikacin; AMP, Ampicillin; AMC, Amoxicillin+ clavulanic acid; CZA, Ceftazidime-Avibactam; CAZ, Ceftazidime; C/T Ceftolozane/ Tazobactam; CIP, Ciprofloxacin; CTX, Cefotaxime; ERT, Ertapenem; FEP, Cefepime; GEN, Gentamicine; MEM, Meropenem; SXT, Trimethoprim/sulfamethoxazole; P/T, Piperacillin/Tazobactam; TGC, tigecycline; COL, Colistin.
